# Is ZFP57 binding to *H19/IGF2*:IG-DMR affected in Silver-Russell syndrome?

**DOI:** 10.1186/s13148-018-0454-7

**Published:** 2018-02-21

**Authors:** Angela Sparago, Flavia Cerrato, Andrea Riccio

**Affiliations:** 1 Dipartimento di Scienze e Tecnologie Ambientali Biologiche e Farmaceutiche, Università degli Studi della Campania “Luigi Vanvitelli”, Caserta, Italy; 2 Istituto di Genetica e Biofisica “Adriano Buzzati-Traverso”, Consiglio Nazionale delle Ricerche CNR, Naples, Italy

**Keywords:** Genomic imprinting, Silver-Russell syndrome, DNA methylation, ZFP57, *H19/IGF2*:IG-DMR deletions, Beckwith-Wiedemann syndrome

## Abstract

**Background:**

Loss of paternal methylation (LOM) of the *H19/IGF2* intergenic differentially methylated region (*H19/IGF2*:IG-DMR) causes alteration of *H19/IGF2* imprinting and Silver-Russell syndrome (SRS). Recently, internal deletions of the *H19/IGF2*:IG-DMR have been associated with LOM and SRS when present on the paternal chromosome. In contrast, previously described deletions, most of which cause gain of methylation (GOM) and Beckwith-Wiedemann syndrome (BWS) on maternal transmission, were consistently associated with normal methylation and phenotype if paternally inherited.

**Presentation of the hypothesis:**

The presence of several target sites (ZTSs) and three demonstrated binding regions (BRs) for the imprinting factor ZFP57 in the *H19/IGF2*:IG-DMR suggest the involvement of this factor in the maintenance of methylation of this locus. By comparing the extension of the *H19/IGF2*:IG-DMR deletions with the binding profile of ZFP57, we propose that the effect of the deletions on DNA methylation and clinical phenotype is dependent on their interference with ZFP57 binding. Indeed, deletions strongly affecting a ZFP57 BR result in LOM and SRS, while deletions preserving a significant number of ZFPs in each BR do not alter methylation and are associated with normal phenotype.

**Testing the hypothesis:**

The generation of transgenic mouse lines in which the endogenous *H19/IGF2*:IG-DMR is replaced by the human orthologous locus including the three ZFP57 BRs or their mutant versions will allow to test the role of ZFP57 binding in imprinted methylation and growth phenotype.

**Implications of the hypothesis:**

Similarly to what is proposed for maternally inherited BWS mutations and CTCF and OCT4/SOX2 binding, we suggest that deletions of the *H19/IGF2*:IG-DMR result in SRS with LOM if ZFP57 binding on the paternal chromosome is affected.

**Electronic supplementary material:**

The online version of this article (10.1186/s13148-018-0454-7) contains supplementary material, which is available to authorized users.

## Background

The imprinted monoallelic expression of the *H19* and *IGF2* genes is controlled by differential methylation of the *H19/IGF2* intergenic differentially methylated region (*H19/IGF2*:IG-DMR) on the maternal and paternal chromosome 11p15.5 [1]. DNA methylation abnormalities of the *H19/IGF2*:IG-DMR cause contrasting growth disorders (i.e., Beckwith-Wiedemann syndrome (BWS, MIM#130650) and Silver-Russell syndrome (SRS, MIM#180860)), if they are present on the maternal or paternal chromosome, respectively [2]. More specifically, 5–10% of BWS cases are caused by gain of methylation (GOM) of the maternal *H19/IGF2*:IG-DMR resulting in biallelic expression of the growth factor *IGF2* and silencing of the growth inhibitor *H19*, while 40–60% of SRS cases are associated with loss of methylation (LOM) of the paternal *H19/IGF2*:IG-DMR leading to *IGF2* suppression and biallelic *H19* expression*.*

We and others have previously demonstrated that GOM is associated with maternal transmission of internal deletions or single nucleotide variants (SNVs) of the *H19/IGF2*:IG-DMR in a subgroup of BWS cases [3–13]. These mutations affect target sites for CTCF (CTSs) and/or OCT4/SOX2 (OTSs/STS) that likely result in methylation of the remaining *H19/IGF2*:IG-DMR sequence on the maternal chromosome and imprinting alteration (Table 1 and Additional file 1: Table S1). These studies also show that paternal transmission of these mutations is consistently associated with normal methylation and clinical phenotype. In apparent contrast with these observations, Abi Habib and co-workers have recently reported new *H19/IGF2*:IG-DMR deletions that are associated with LOM and SRS, when paternally inherited [14].

**Table 1:**
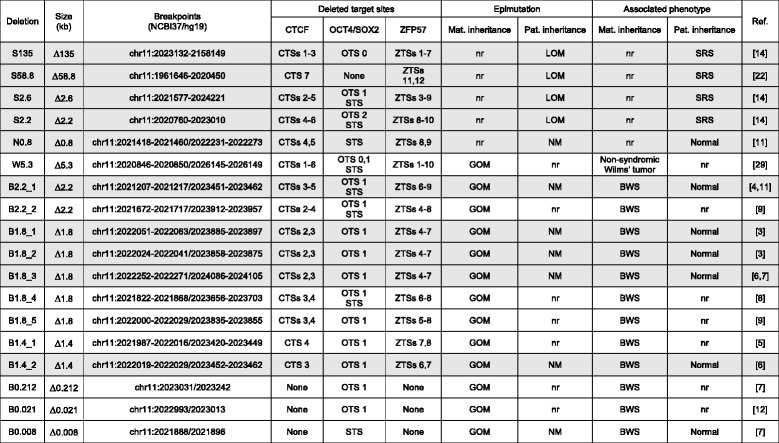
List of all *H19/IGF2*:IG-DMR deletions [3–9, 11, 12, 14, 22, 29]

Only deletions involving ZTSs, with reported phenotype on paternal transmission (in gray shades), were considered for this study

*CTSs* CTCF target sites, *OTSs* OCT4 target sites, *STS* SOX2 target site, *ZTSs* ZFP57 target sites, *GOM* gain of methylation, *LOM* loss of methylation, *NM* normal methylation, *nr* not reported, *BWS* Beckwith-Wiedemann syndrome, *SRS* Silver-Russell syndrome

## Presentation of the hypothesis

The *H19/IGF2*:IG-DMR acquires DNA methylation in male germ cells and maintains it on the paternal allele of somatic cells throughout development despite the intense epigenetic reprogramming occurring post-fertilization [15, 16]. In mouse embryos and ESCs, maintenance of methylation at the orthologous *H19/Igf2*:IG-DMR as well as other imprinted DMRs is ensured by binding of the zinc-finger protein ZFP57, which is needed to recruit a number of heterochromatin-associated factors, including the corepressor KAP1, DNA methyltransferases, and histone methyltransferases [17–19]. Differently from CTCF and OCT4/SOX2 that bind the unmethylated maternal allele, ZFP57 recognizes a methylated TGCCGC motif on the paternal allele, present in multiple copies in the *H19/Igf2*:IG-DMR [20].

**Fig. 1:**
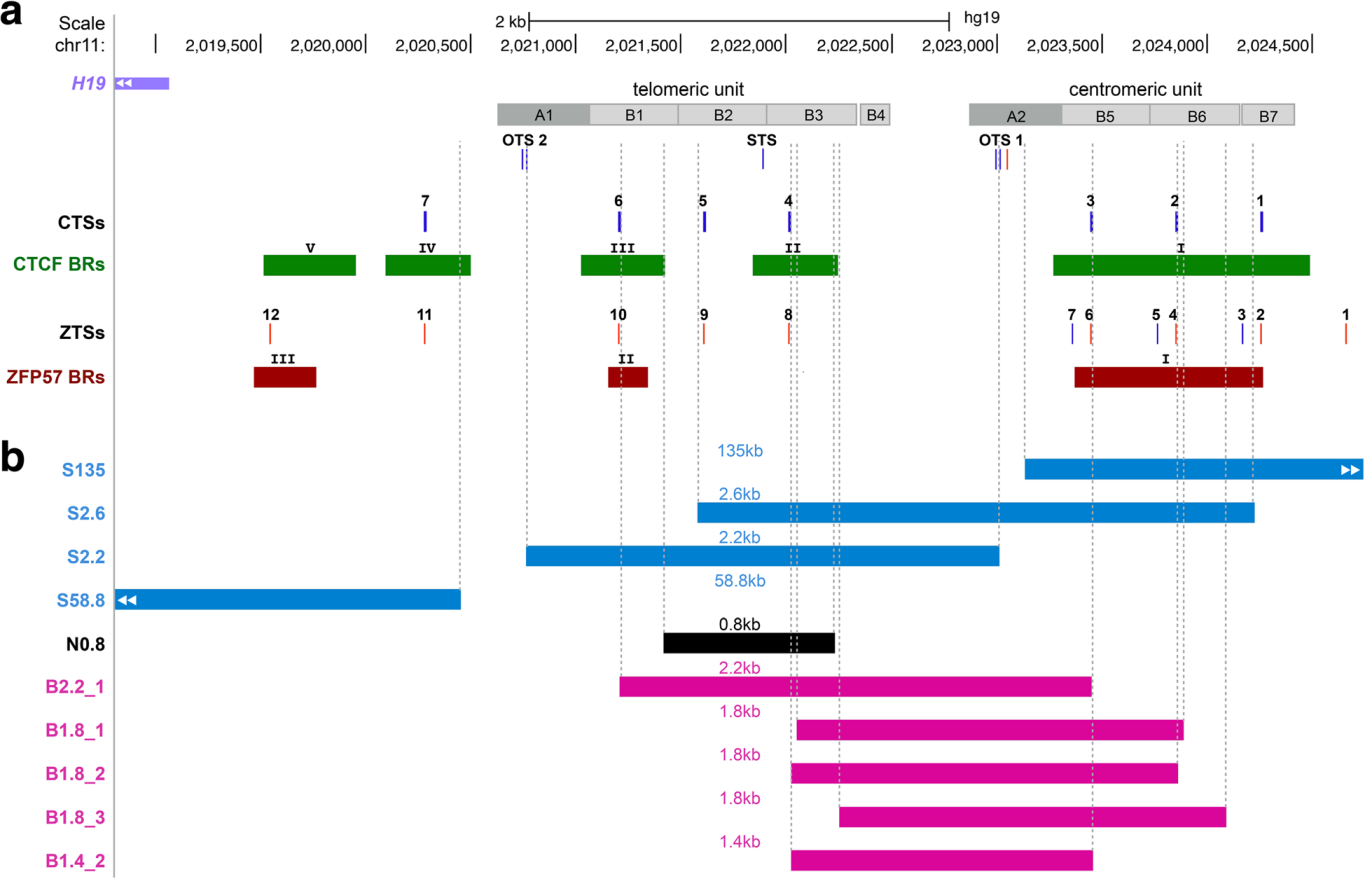
Paternal *H19/IGF2*:IG-DMR deletions and ZFP57 binding. **a** Schematic representation of the *H19/IGF2*:IG-DMR. The genomic region corresponds to GRCh37/hg19 chr11:2,018,812–2,024,740 [28] on the UCSC Genome Browser. The A- and B-type repetitive modules are represented as gray boxes. Target sites for OCT4 (OTSs), SOX2 (STS), CTCF (CTSs), and ZFP57 (ZTSs) are indicated by vertical bars; motif sequences present in the forward strand are in red, those present in the reverse strand are in blue. The CTCF BRs (transcription factor ChIP-seq, ENCODE data) are indicated as green bars. The ZFP57 BRs demonstrated in HEK293T cells (GSM2466451 [21]) are indicated as brown bars. **b** Paternally inherited *H19/IGF2*:IG-DMR deletions. Deletions associated with SRS and LOM on paternal transmission [14, 22] are represented in blue; the deletion associated with normal methylation and normal phenotype on paternal transmission [11] is in black; deletions associated with BWS and GOM on maternal transmission and normal phenotype on paternal transmission [3, 4, 6, 7, 11] are in pink. Breakpoints, molecular, and phenotypic effects of all the deletions are reported in Table 1

The human *H19/IGF2*:IG-DMR has a repetitive structure and includes two units of tandem repeats each composed of shorter repetitive modules (Fig. 1a). Twelve potential ZFP57 target sites (ZTSs) are present within the DMR, either as single isolated hexameric motifs or as closely spaced doublets of hexamers (Additional file 1: Table S1). In particular, the telomeric unit includes three singlets and the centromeric unit three doublets. Notably, ZTS doublets appear to be particularly important for ZFP57 binding in the mouse [20]. The genomic profile of ZFP57 binding, which has recently been obtained in a human cell line, is consistent with the location of the ZTSs [21]. Indeed, three ZFP57 binding regions (ZFP57 BRs) were demonstrated in the *H19/IGF2*:IG-DMR, with the largest one corresponding to the three ZTS doublets of the centromeric unit (Fig. 1a). Based on the comparison of the *H19/IGF2*:IG-DMR deletions (Fig. 1b and Table 1) with the ZFP57 binding profile and target sites, we propose that ZFP57 binding can explain the differential effects of these mutations on paternal transmission and that only the deletions interfering with ZFP57 result in SRS.

The three deletions reported by Abi Habib et al. (S135, S2.6, and S2.2) affect different portions of the DMR (Fig. 1b). S135 eliminates the ZFP57 BR-I and all the ZTSs (2–7) of the centromeric unit. S2.6 also affects BR-I with ZTSs 3–7, and in addition, removes two ZTSs (8, 9) from the telomeric unit. S2.2 abolishes BR-II with all the ZTSs (8–10) of the telomeric unit. All three deletions are associated with LOM, but a more severe hypomethylation was reported for S135 and S2.6, suggesting that the centromeric unit plays a major role in methylation maintenance [14]. The presence of a large ZFP57 binding region (BR-I) and a higher number of ZTSs within this region (Fig. 1a) is consistent with this observation.

In 2011, Grønskov and co-workers [22] described another SRS case with a paternal deletion (S58.8) associated with LOM of the *H19/IGF2*:IG-DMR (Fig. 1b). In addition to the *H19* promoter, gene, and telomeric enhancers, this mutation removed the ZFP57 BR-III with ZTSs 11 and 12, indicating that also this region is involved in methylation maintenance (Fig. 1a, b).

How to explain the normal phenotype associated with the other deletions? Why they do not cause LOM on paternal transmission? N0.8 does not affect any ZFP57 BR (Fig. 1b). B2.2_1, B1.8_1–3, and B1.4_2 only partially affect either BR-I or BR-II. In particular, differently from the SRS-associated deletions, they never include the ZTS doublet 2–3 of BR-I and ZTS 10 of BR-II, which may be sufficient for ZFP57 binding.

There is a possible limitation in our hypothesis. ZFP57 BRs were demonstrated in a transformed cell system (HEK293T cells) overexpressing the human protein, and they might not faithfully represent all the BRs of the endogenous ZFP57 in its biological relevant tissues/developmental time points. However, if we take into consideration the ZTSs not included in BRs, an important role of ZTSs 8 and 9 is excluded by normal methylation of the N0.8 deletion, while a possible contribution of ZTS 1 and ZTS 11 to ZFP57 binding would not affect our hypothesis (Fig. 1a, b). A further possible criticism in our hypothesis concerns the normal methylation of the *H19/IGF2*:IG-DMR in transient neonatal diabetes mellitus 1 (TNDM1, OMIM 601410) patients with loss of function mutations of ZFP57 and multi-locus imprinting disturbances (MLID) [23, 24]. However, it is possible that this methylation pattern results from phenotypic selection of affected loci and that this might not necessary represent all the loci regulated by ZFP57 in humans. Moreover, although no ZFP57 mutation has been demonstrated, a defect of this gene in SRS cannot be excluded because only a few cases with MLID have been screened, so far [25, 26].

### Testing the hypothesis

We recently described a transgenic mouse line in which the endogenous mouse *H19/Igf2* IG-DMR was replaced by the orthologous human sequence (chr11:2,019,934–2,024,611) including ZTSs 2–11 [27]. While its function was conserved upon maternal transmission, the humanized locus was not properly methylated in sperms and methylation not maintained in somatic cells on paternal transmission. We now observe that this transgene was lacking BR-III that could be necessary for methylation maintenance. To test this hypothesis, we propose to generate a new knock-in mouse carrying the complete human *H19/IGF2*:IG-DMR sequence with all its ZFP57 BRs, which we expect to establish and maintain the imprinted methylation correctly. Similarly, mutants of the most relevant ZFP57 target sites (ZTSs 2–3, ZTS 10, and ZTS 12) can be generated to test their specific role in SRS etiology.

## Implications of the hypothesis

Our hypothesis implies that the effect of *H19/IGF2*:IG-DMR deletions depends on two factors: the parental origin of the mutation and the transcription factors whose binding is affected. So far, maternal deletions, which are all associated with BWS, have been proposed to alter the binding of CTCF and/or OCT4/SOX2 resulting in DMR hypermethylation. Conversely, we propose that paternal deletions are associated with SRS when ZFP57 binding is affected and this happens when one of the three ZFP57 BRs is lost. Since the role of some ZTSs appears critical for methylation maintenance, SNVs affecting these binding sites may be present in deletion-negative SRS cases with *H19/IGF2*:IG-DMR LOM.

## Additional file

Additional file 1: **Table S1.** List of CTCF, OCT4/SOX2 and ZFP57 binding regions and potential target sites in the *H19/IGF2*:IG-DMR. Genomic positions of the binding regions (if demonstrated) and potential target sites and motifs of all these factors have been listed. (PDF 86 kb)

## Abbreviations

BR: Binding region
BWS: Beckwith-Wiedemann syndrome
CTS: CTCF target site
ESC: Embryonic stem cell
GOM: Gain of methylation
*H19/IGF2*:IG-DMR *H19/IGF2*: Intergenic differentially methylated region
LOM: Loss of methylation
MLID: Multi-locus imprinting disturbances
NM: Normal methylation
nr: Not reported
OTS: OCT4 target site
SNV: Single nucleotide variation
SRS: Silver-Russell syndrome
STS: SOX2 target site
TNDM1: Transient neonatal diabetes mellitus 1
ZTS: ZFP57 target site
