# Supplementary material for: Is ZFP57 binding to H19/IGF2:IG-DMR affected in Silver-Russell syndrome?
Source: Clin Epigenetics. 2018 Feb 21;10:23. doi: 10.1186/s13148-018-0454-7 (PMC5822596; doi:10.1186/s13148-018-0454-7)
Supplement: Supplementary file 1 — Table S1. List of CTCF, OCT4/SOX2 and ZFP57 binding regions and potential target sites in the H19/IGF2:IG-DMR. Genomic positions of the binding regions (if demonstrated) and potential target sites and motifs of all these factors have been listed. (PDF 86 kb) [file 13148_2018_454_MOESM1_ESM.pdf]

Table S1 List of CTCF, OCT4/SOX2 and ZFP57 binding regions and potential target sites in the *H19/IGF2*:IG-DMR

Binding Regions	Ref.	Genomic Position (NCBI37/hg19)	Target Site	Binding motif	Ref.	Genomic Position (NCBI37/hg19)	Strand
CTCF BR-I	ENCODE	chr11:2023265-2024482	CTS 1	CCGCGCGGCGGCAG	[30,31]	chr11:2024249-2024262	-
			CTS 2	CCGCGCGGCGGCAG		chr11:2023844-2023857	-
			CTS 3	CCGCGCGGCGGCAG		chr11:2023443-2023456	-
CTCF BR-II	ENCODE	chr11:2021843-2022240	CTS 4	CCGCGTGGCGGCAG		chr11:2022010-2022023	-
nr		nr	CTS 5	CTGCGCGGCGGCAG		chr11:2021604-2021617	-
CTCF BR-III	ENCODE	chr11:2021026-2021421	CTS 6	CCGCGCGGCGGCAG		chr11:2021198-2021211	-
CTCF BR-IV	ENCODE	chr11:2020097-2020497	CTS 7	CCGAGAGGCGGCAG		chr11:2020275-2020288	-
CTCF BR-V	ENCODE	chr11:2019516-2019958	nr	nr	nr	nr	nr
nr	nr	nr	OTS 0*	ATGCAAAT<br>AAGCAAAT	[32]	chr11:2025855-2025862<br>chr11:2025836-2025843	-<br>-
nr	nr	nr	OTS 1	ATGTTAAT<br>ATGCTAAT<br>ATGCTAAA		chr11:2023043-2023050<br>chr11:2023013-2023020<br>chr11:2022992-2022999	+<br>-<br>-
nr	nr	nr	OTS 2	GTGCTAAT<br>ATGCTAAA		chr11:2020762-2020769<br>chr11:2020741-2020748	-<br>-
nr	nr	nr	STS	CATTCATG	[7]	chr11:2021888-2021895	-
nr	nr	nr	ZTS 1	TGCCGC	This study	chr11:2024654-2024659	+
ZFP57 BR-I	[21]	chr11:2023368-2024263	ZTS 2	TGCCGC		chr11:2024250-2024255	+
			ZTS 3	TGCCGC		chr11:2024165-2024170	-
			ZTS 4	TGCCGC		chr11:2023845-2023850	+
			ZTS 5	TGCCGC		chr11:2023759-2023764	-
			ZTS 6	TGCCGC		chr11:2023444-2023449	+
			ZTS 7	TGCCGC		chr11:2023358-2023363	-
nr	nr	nr	ZTS 8	TGCCGC		chr11:2022011-2022016	+
nr	nr	nr	ZTS 9	TGCCGC		chr11:2021605-2021610	+
ZFP57 BR-II	[21]	chr11:2021157-2021342	ZTS 10	TGCCGC		chr11:2021199-2021204	+
nr	nr	nr	ZTS 11	TGCCGC		chr11:2020276-2020281	+
ZFP57 BR-III	[21]	chr11:2019473-2019764	ZTS 12	TGCCGC		chr11:2019545-2019550	+

CTSs, CTCF target sites; OTSs, OCT4 target sites; STS, SOX2 target site; ZTSs, ZFP57 target sites; nr, not reported; +, forward strand; -, reverse strand.
*This site flanks centromerically the *H19/IGF2*:IG-DMR.

ADDITIONAL REFERENCES:

30. Hark AT, Schoenherr CJ, Katz DJ, Ingram RS, Levorse JM, Tilghman SM. CTCF mediates methylation-sensitive enhancer-blocking activity at the *H19/Igf2* locus. Nature. 2000;405(6785):486-89.

31. Bell AC, Felsenfeld G. Methylation of a CTCF-dependent boundary controls imprinted expression of the *Igf2* gene. Nature. 2000;405(6785):482-85.

32. Hori N, Nakano H, Takeuchi T, Kato H, Hamaguchi S, Oshimura M, et al. A dyad oct-binding sequence functions as a maintenance sequence for the unmethylated state within the *H19/Igf2*-imprinted control region. J Biol Chem. 2002;277(31):27960-67.
